# The Incidence of Spontaneous Abortion in Mothers with Blood Group O Compared with other Blood Types

**Published:** 2012

**Authors:** Mohammad Hassanzadeh-Nazarabadi, Sahar Shekouhi, Najmeh Seif

**Affiliations:** *Faculty of Medicince, Department of Medical Genetics, Mashhad University of Medical Sciences, Mashhad, Iran.*

**Keywords:** Spontaneous abortion, ABO blood group, incompatibility

## Abstract

Although ABO incompatibility between mother and fetus has long been suspected as cause of spontaneous abortion in man, its precise contribution has not been completely resolved. In spite of reports in which the incompatible mating was recognized to be a cause of habitual abortion, and which eventually results in infertility or a reduction in the number of living children compared with the number in compatible matings, such effects were not observed in other studies. The aim of this review article was to show some evidence of relationship between ABO incompatibility and spontaneous abortion.

In 1900 Karl Landsteiner reported a series of tests, which identified the ABO blood group system. This is the only blood group in which antibodies are constantly, predictably, and naturally present in the serum of people who lack the antigen. ABO compatibility between mother and fetus is crucial (1).

**Abortion**

Spontaneous abortion also known as miscarriage, refers to a pregnancy that ends spontaneously before the fetus has reached a viable gestational age (2-4).

**Relationship between ABO Blood Group and Spontaneous Abortion**

Sixty-two articles in relation to ABO incompatibility and abortion have been published so far and one of the latest was published in 2011.

Shortly after the ABO blood groups were discovered, attention was directed toward the possibility of harmful effects when mother and fetus have different blood groups. As early as 1905 A. Dienst suggested that toxemia of pregnancy might be due to the transfusion of ABO-incompatible fetal blood into the mother. This was not substantiated, and the problem of ABO interaction between mother and fetus was largely overshadowed by the more dramatic effects of Rh incompatibility leading to Rh hemolytic disease. Nevertheless, work has continued on ABO interactions, and particularly on ABO incompatibility in which the maternal and paternal blood groups are such that the father would be unable to donate blood to the mother. Thus, although the blood groups are different, an A mother and an O father would be ABO-compatible, but an O mother and an A father would be defined as an ABO incompatible mating. A further complication is that a father with blood group A may be homozygous AA or heterozygous AO, and thus an ABO-incompatible mating in which the father is A and the mother O may produce a compatible O or an incompatible A fetus.

During the second half of the 20th century, many papers have been published reporting the spontaneous abortion due to blood group incompatibility and the operation of natural selection in the human ABO blood group system. The purpose of this article is to review those and conclude whether the spontaneous abortion is a result of incompatible ABO blood group or not.

## Materials and Methods

We have searched the keywords "spontaneous abortion", "ABO blood group" and "incompatibility" in two scientific databases; Pubmed and Google scholar. We limited the search to articles published from 1950 till now. Then the articles were sorted by relevance to the subject. Most relevant papers were chosen to be reviewed.

**Evidence of Relationship between ABO Blood Group and Spontaneous Abortion**

In 1972 Takno and Miller studied the ABO incompatibility as a cause of spontaneous abortion. In this study, 229 cases of spontaneous abortion not exceeding 20 weeks of gestation were observed at the Vancouver General Hospital. The maternal blood type was distributed as follows: O, 52. 0%; A, 37. 1 %; B, 9.2%; AB, 1.7%. The incidence of O type mothers who are most likely to have a spontaneous abortion, if maternal-fetal ABO incompatibility does play a role in this phenomenon, was considerably higher than in the general population of British Columbia (44.5%).

In order to observe the exact maternal-fetal blood group relations in each case of spontaneous abortion, ABO blood types of the abortuses were tested by the mixed agglutination technique. From the 78 fetuses examined, 35 (44.7%) were found to be incompatible with the mother. This frequency of incompatible cases was significantly higher than that expected (p<0.01). There was no apparent interaction between the ABO and Rh incompatibilities. Finally, they concluded that ABO incompatibility is at least one of the major causes of spontaneous abortion in the gestational stages between 35-40 days to 135 days. The results showed that the incompatible conspectuses were more likely to be aborted than the compatible ones (5).

The method used in this study is a more powerful method of analysis than simply determining the ABO status of the parents, because an ABO incompatible mating does not necessarily produce an ABO incompatible fetus (6). In the same year, Clarke studied the practical effects of blood group incompatibility between mother and fetus, named early abortion as an effect of blood group incompatibility and mentioned that there is no doubt that ABO incompatibility between early abortuses and the mother is a factor in causing abortion (6).

Several other studies have also been reported which examined the relationship between ABO incompatibility and reproductive failure. In 1984 Schaap et al. had a parallel analysis of the frequencies of blood-group combinations in mother/child pairs and of fetal losses which revealed two discrepancies: (1) although the combination mother B/child AB is rarer than expected, no increased rate of previous spontaneous abortions can be demonstrated in these women; and (2) even though blood-group-0 mothers with blood-group-B infants have had more previous spontaneous abortions than other women in their sample, the combination mother O/child B is not rarer than expected. They also interpreted that prenatal selection associated with ABO incompatibility may operate at various stages for fertilization through pregnancy (7).

In 2001 Bottini et al. studied the differential effects of ABO incompatibility on fertility through a comparative analysis of couples with recurrent spontaneous abortion (RSA) and healthy mothers in two populations (Rome and Sassari).

In this study, ABO phenotype has been determined in 5180 healthy mothers and their newborn babies in the population of Sassari (Sardinia) and in 1359 healthy women who have just given birth in the population of Rome. Mother-newborn joint ABO distribution in healthy mothers was compared with wife-husband joint ABO distribution in RSA couples. Distortions from expected distribution were evaluated by symmetry analysis (8).

In 2004, 79 couples suffering from repeated abortion have been investigated by Malekasgar et al. for the ABO blood incompatibility as a cause of abortion. The results showed that there is a clear increase in the number of individuals for blood groups A and AB in patients with repeated abortion, and this factor may isneeded to be paid more attention to in more investigations. It is possible that incompatibility of the antigens present in red blood cell membrane of husband/wife may play some role in abortion (9).

In another study performed in India in 2009 the couple combinations having O type wives, A or B type husbands showed maximum fetal loss. As far as abortion is concerned it is higher in A type husband and O type wife and stillbirths are higher in couple combination A type husband and B type wife (10).

The most recent study performed by Mohanty and Das in India discusses the effects of natural selection on four population groups of Orisson in the form of differential fertility and mortality as a consequence of ABO incompatibility. The incidence of abortion, stillbirth and post natal mortality are marginally higher among incompatible couples than compatible couples without revealing significant statistical difference except in Mohanta (11).

In 2004, Pourjafari et al. performed a study on the frequencies of antigens and their alleles of ABO and Rhesus blood systems in a group of women with recurrent abortion, as compared with the general population in Hamedan, Iran. In ABO system, the most frequent blood types were O, A, B and AB respectively in case group. The frequent alleles were O, A, B, respectively. The frequencies of alleles were not statistically different in two groups. There fore it can be concluded that the results of ABO blood type had no role in recurrent abortion. Also, the frequent allele in Rhesus system was "D". The frequency of D allele in two groups was statistically different (p=0.05). Finally, they demonstrated that Rh negative was a risk factor for recurrent abortion in case population (12).

In a study performed in 2004 by Sharma and Kapoor the possible differential effects of ABO blood group materno-paternal (fetal) incompatibility on completed reproductive performance were investigated on a sample of 100 couples (100 fathers and 100 mothers) from three villages in the Jind district of Haryana state, India (13).

The average number of living children in the compatible matings (4.64) was higher than the incompatible ones (4.18). With reference to individual ABO matings, the index of relative fertility (Irf) was the highest in A x AB followed by B x A type of incompatible matings. No decrease in live births in O x A and O x B incompatible matings was observed compared with their reciprocal compatible ones, i.e. A x O and B x O matings as had been hypothesized in previous studies. The total pregnancy wastage was substantially higher in ABO-incompatible matings (24.59%) than compatible matings (8.45%). The study supports the hypothesis that selection is operative at the ABO locus (15). Another evidence of the relationship between ABO blood group incompatibility and spontaneous abortion is the study performed by Bandyopadhyay et al. in 2011. In the study of 124 spontaneous abortions occurring during the first 16 weeks of gestation, simultaneous karyotyping and ABO blood grouping of 148 of the parents has been carried out. In 80 of the 124 chromosome-analyzed aborted fetuses, the ABO blood groups were determined by the mixed cell agglutinating reaction in fetal tissue. Among the aborted fetuses with normal karyotype, a significantly higher (P < 0.05) frequency of ABO incompatibility was found in couple combination in comparison with the couple combination of the parents of the newborns. Furthermore, the distribution ABO blood group alleles of the fetuses deviated significantly from those of newborns (P < 0.05) in terms of a higher A alleles among the fetus. Finally, it is concluded that the ABO incompatibility between the couples is likely to be a risk factor for early spontaneous abortions and also the heterozygote selection of ABO blood group genotypes (14).

**Blood group incompatibility is more frequent in couples with recurrent abortion than fertile couples**

Blood group antigens are markers on surface-exposed red cell proteins or the sugar moiety of glycoproteins or glycolipids (15-17).

Four phenotypes are usually seen in ABO blood group antigens, which are expressed by three alleles. These phenotypes are A, B, AB and O. If a person has inherited allele A (AA or AO), N-acetylgalactosaminyl transferase adds N-acetylgalactosame to protein H. If a person has inherited allele B (BO or BB), galactosyl transferase adds D-galactose to protein H. If a person has inherited both alleles, A and B (AB), both enzymes act and make blood group AB with two antigens (18-22).

Blood groups study revealed that an incompatibility of the blood groups can influence reproduction. Moreover, the mortality of mothers in pregnancy was considerably higher in couples who had blood group incompatibility (23).

The widely repeated delivery of abnormal newborn and stillbirth were more frequent in couples with incompatible blood group than others (24). Infertility could occur in couples with incompatible blood groups (25). However, some studies disagreed with this idea (26). Acceptance of the fetus, which expresses paternally inherited alloantigens by the mother during pregnancy is an exclusive example of how the immune system alters a destructive alloimmune response to a state of tolerance (27-28).

ABO incompatibility happens in 20 to 25% of pregnancies and hemolytic disease develops in about 10% of such offspring. After delivery, neonates are admitted with high total serum bilirubin mostly because of ABO incompatibility (29-30).

Isoimmune hemolytic disease of the newborn are mostly caused by ABO incompatibility. This hemolytic disease is clinically milder than Rh incompatibility, but severe hemolysis occasionally occurs, and some cases need exchange transfusion (31).

ABO incompatibility is one of the most common reasons of bilirubin encephalopathy, and in the uterus this can cause impaired pregnancy and miscarriage (32-34).Positive direct coombs’ test and positive family history of neonatal jaundice or previous abortion strongly predict ABO incompatibility (34-35).

## Conclusions

 ABO incompatibility occurs in 20% of pregnancies, but only 20% of these develop hemolytic disease which is milder than Rh incompatibility and can lead to abortion in the uterus (36-38).

The blood groups are different, an A mother and an O father will be ABO-compatible, but an O mother and an A father will be defined as an ABO incompatible mate. A further complication is that a father with blood group A may be homozygous (AA) or heterozygous (AO), and thus an ABO-incompatible mating in which the father is A and the mother O may produce a compatible O or an incompatible A fetus. Finally the assessment of couples’ blood group with abortion indicate that the incompatibilities are considerably more common than in normal couples (39).

## References

[1] Hosoi E (2008). Biological and clinical aspects of ABO blood group system. J Med Invest.

[2] Regan L, Rai R (2000). Epidemiology and the medical causes of miscarriage. Best Pract Res Cl Ob.

[3] Abc.

[4] (1972). ABO blood groups and abortion. Br Med J.

[5] Takano K, Miller JR (1972). ABO incompatibility as a cause of spontaneous abortion: evidence from abortuses. J Med Genet.

[6] Clarke CA (1972). Practical effects of blood group incompatibility between mother and fetus. Br Med J.

[7] Schaap T, Shemer R, Palti Z et al (1984). ABO incompatibility and reproductive failure. I. Prenatal selection. Am J Hum Genet.

[8] Bottini N, Meloni GF, Finocchi A (2001). Maternal-fetal interaction in the ABO system: A comparative analysis of healthy mothers and couples with recurrent spontaneous abortion suggests a protective effect of B incompatibility. Hum Biol.

[9] Malekasgar AM (2004). ABO blood group prevalence in spontaneously repeated abortion. Turk J Haematol.

[10] Soni N, Mukherjee BM (2009). A Study on Foetal Wastage and ABO Blood Groups Incompatibility among the Gondsof Garriyaband, Chhattisgarh, India. Anthropologist.

[11] Mohanty R, Das PK (2010). A Search for Operation of Natural Selection in ABO Blood Groups: Evidences from Four Ethnic Groups of Orissa. Anthropologist.

[12] Pourjafari H, Hashemzadeh Chaleshtori M, Arab M (2004). Frequencies of antigens and their allels from ABO & RH blood types in a group of women with two or more abortions. Scientific Journal of Hamadan University of Medical Sciences.

[13] Sharma K, Kapoor R (2004). Abo blood groups and completed reproductive performance of rural Haryanavi couples: analysing measures of selection intensities. J Biosoc Sci.

[14] Bandyopadhyay AR, Chatterjee D, Chatterjee M et al (2011). Maternal Fetal Interaction in the ABO System: A Comparative Analysis of Healthy Mother and Couples with Spontaneous Abortion in Bengalee Population. Am J Hum Biol.

[15] Marsh WL (1990). Biological roles of blood group antigens. Yale J Biol Med.

[16] Badet J (1976). Serum glycosyltransferase activity associated with antigen biosynthesis in blood groups A and B. Study of normal B group and cis AB group subjects. Rev Fr Transfus Immunohematol.

[17] Lloyd KO, Kabat EA, Licerio E (1968). Immunochemical studies on blood groups. 38. Structures and activities of oligosaccharides produced by alkaline degradation of blood-group Lewis-a substance. Proposed structure of the carbohydrate chains of human blood-group A, B, H, Le-a, and Le-b substances. Biochemistry.

[18] Race C, Ziderman D, Watkins WM (1968). An alpha-d-galactosyltransferase associated with the blood-group B character. Biochem J.

[19] Shen L, Grollman EF, Ginsburg V (1968). An enzymatic basis for secretor status and blood group substance specificity in humans. Proc Natl Acad Sci U S A.

[20] Watkins WM, Morgan WT (1959). Possible genetical pathways for the biosynthesis of blood group mucopolysaccharides. Vox Sang.

[21] Yoshida A, Dave V, Branch DR (1982). An enzyme basis for blood type A intermediate status. Am J Hum Genet.

[22] Morgan WT, Watkins WM (1969). Genetic and biochemical aspects of human blood-group A-, B-, H-, Le-a- and Le-b-specificity. Br Med Bull.

[23] Lorigan PC, Sharma S, Bright N (2000). Characteristics of women with recurrent molar pregnancies. Gynecol Oncol.

[24] Berberovic L, Redzic A, Sosic B (2004). Impact of ABO blood groups on the fertility of different parental pairs. Bosn J Basic Med Sci.

[25] Omu AE, Al-Mutawa M, Al-Qattan F (1998). ABO blood group and expression of antisperm antibodies in infertile couples in Kuwait. Gynecol Obstet Invest.

[26] Bakkeheim E, Bergerud U, Schmidt-Melbye AC (2009). Maternal IgG anti-A and anti-B titres predict outcome in ABO-incompatibility in the neonate. Acta Paediatr.

[27] Guleria I, Sayegh MH (2007). Maternal acceptance of the fetus: true human tolerance. J Immunol.

[28] Nasseri F, Mamouri GA, Babaei H (2006). Intravenous immunoglobulin in ABO and Rh hemolytic diseases of newborn. Saudi Med J.

[29] Kneib MT, Hamon I, Miton A (2002). Management of severe neonatal Rh disease following in utero exchange transfusion: towards a new strategy. Arch Pediatr.

[30] Wallerstein H (1949). The management of hemolytic disease of the fetus and newborn infant. Acta Haematol.

[31] Wikman A, Edner A, Gryfelt G (2007). Fetal hemolytic anemia and intrauterine death caused by anti-M immunization. Transfusion.

[32] Lurie S, Sigler E, Weissman A (1998). Association of the Lewis blood-group phenotype with infertility in women. Int J Fertil Womens Med.

[33] Mamouri GA, Babaei H (2002). ABO –Hemolytic disease of the newborn. Medical journal of Mashhad University of medical sciences.

[34] Ogunlesi TA, Dedeke IO, Adekanmbi AF (2007). The incidence and outcome of bilirubin encephalopathy in Nigeria: a bi-centre study. Niger J Med.

[35] Richon J, Streiff F, Genett B (1970). Value of the Coombs-Bromeline test in ABO feto-maternal blood incompatibilities. Bull Fed Soc Gynecol Obstet Lang Fr.

[36] Scid Vidal J, Elies Fibla E (2000). Immunohematologic study of ABO hemolytic disease. An Esp Pediatr.

[37] Gao XY, Huang H, Li LD (2009). Hemolytic disease of neonates due to anti-M: report of one case and review of reports of 21 cases. Zhonghua Er Ke Za Zhi.

[38] Marwaha N, Dhawan H, Thakral B (2009). Severe ABO hemolytic disease of newborn with a positive direct antiglobulin test. Indian J Pathol Microbiol.

[39] Sarici SU, Alpay F, Yesilkaya E (2002). Hemolytic disease of the newborn due to isoimmunization with anti-E antibodies: a case report. Turkish J Pediatr.

[40] Thilo EH, Rosenburg AA, Hay WW, Levin MJ, Sondheimer JM (2007). The newborn infant: common problems in the newborn infant: neonatal jaundice. Current pediatric diagnosis and treatment.

